# Cure kinetic study of methacrylate-POSS copolymers for ocular lens

**DOI:** 10.1007/s40204-017-0074-x

**Published:** 2017-10-25

**Authors:** F. Shokrolahi, M. Zandi, P. Shokrollahi, M. Atai, E. Ghafarzadeh, M. Hanifeh

**Affiliations:** 1 Biomaterials Department, Iran Polymer and Petrochemical Institute, Pazhoohesh Blvd, Tehran-Karaj Hwy, 1497713115 Tehran, Iran; 2 Science Department, Iran Polymer and Petrochemical Institute, Pazhoohesh Blvd, Tehran-Karaj Hwy, 1497713115 Tehran, Iran; 3 Lassonde School of Engineering, York University, 4700 Keele Street, 11 Arboretum Ln, ON M3J 1P3 Toronto, Canada

**Keywords:** Acrylate-POSS, Polyhedral oligomeric silsesquioxane, Kinetic study, Copolymerization, Contact lens

## Abstract

**Abstract:**

The physical, mechanical and biological properties of multicomponent acrylate-based hard lenses are directly influenced by degree of conversion achieved during copolymerization. In this research, polyhedral oligomeric silsesquioxane (POSS) acrylate is introduced into the polymer backbone in combination with hydroxyethyl methacrylate, dimethyl itaconate, methyl methacrylate, 3-(trimethoxysilyl) propyl methacrylate and triethylene glycol dimethacrylate in free-radical bulk polymerization. Kinetics of curing process was investigated by two techniques: differential scanning calorimetry and FTIR spectroscopy. Reaction kinetics in free-radical bulk polymerization of the system was studied by isothermal DSC performed at 65, 75, 85, and 95 °C using different quantities of initiator. Three compounds were prepared in different concentrations (0.2, 0.4 and 0.6 mol%) of 2,2′-azobisisobutyronitrile as initiator. Conversion rate was calculated as a function of time using data obtained from DSC measurements. The kinetic parameters of the reaction such as reaction constants, reaction orders and activation energies were obtained from the isothermal DSC data according to the autocatalytic model developed by Kamal and Sourour. The results showed that the experimental values were in good agreement with theoretically estimated values and our results may suggest that the polymerization reaction of this system is well described by Kamal’s model. Cytotoxicity results, performed on extracts 28 days after PBS incubation, showed no toxicity of the materials extracted from the lenses indicating that they can be considered as safe materials in ocular lens applications. The viability and proliferation of L929 fibroblast cells in extracting media were followed by 3-[4,5-dimethylthiazol-2-yl]-2,5-diphenyltetrazolium bromide (MTT) and they may have a great potential as ideal supporting lens in people who suffer from keratoconus disease.

**Graphical abstract:**

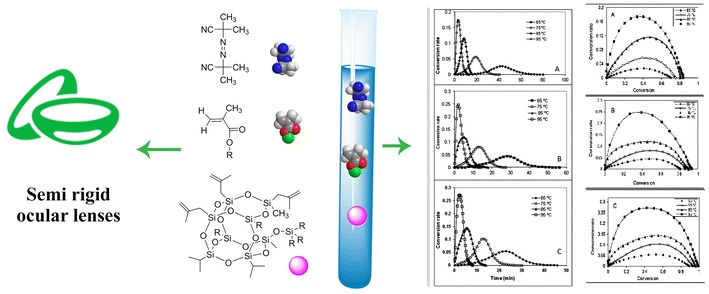

## Introduction

There are many ophthalmic devices, especially designed for eye disorders to provide the best possible vision. Ocular lenses which are considered as medical devices can be worn to correct vision, cosmetic or therapeutic purposes. There are three types of lenses: rigid, soft and hybrid (semi-rigid) lenses due to selected materials and also final applications (Mannis et al. 2003).

Semi-rigid gas-permeable lenses are the primary candidates for correcting the impaired vision arising from deformed cornea. In one therapy methodology offered by Boston (USA), over-sized hard contact lenses of appropriate geometry are made of highly gas-permeable hard plastics that allow oxygen in reach the cornea. The original gas-permeable hard plastic lenses are mainly made of silicone acrylate-based monomers and supplied as a clear dome-shape, approximately the size of a nickel. The device fits under the eyelid overlaying the damaged cornea while leaving a space from the eye that is filled with sterile saline to provide a lubricating pool of artificial tear to the eye. Therefore, the key element of such treatments is the initial hard plastic from which the final device is made according to the patient’s eye characteristics (Rathi et al. 2011). To fabricate biocompatible materials for ocular lenses, it is vital to consider polymerization reaction as well as the starting materials. In this research, a multicomponent acrylate-based copolymer especially designed in ocular lens application is developed.

One of the most interesting techniques for lens preparation is bulk polymerization of multi-functional acrylic-based monomers, because a variety of polymerization methods and systems are achievable. Free-radical bulk polymerization of vinyl monomers, characterized by auto-acceleration, has been widely investigated (Peinado et al. 2002; Wang and Hutchinson 2010). Putting efforts on such investigations is rational because of fabricating the biocompatible materials, is vital to deeply understand and take control over the polymerization steps. The magnitude of such a realization and familiarity is more palpable when considering that the synthesized polymer is being utilized in manufacturing of biomedical devices. Biocompatibility and physical/mechanical properties of the products are greatly influenced by the approach taken to control the reaction. Heat of reaction is also directly linked with the reaction rate (Lee and Lee 1994). It is noticeable that free-radical polymerization of acrylic monomers is highly exothermic; when the reaction temperature is not controlled, it results in undesirable physical and mechanical properties (Vinnik and Roznyatovsky 2004; Woo and Seferis 1990). On the other hand, the presence of any residual unreacted monomer(s) causes some problems for the exposed cells, namely cytotoxicity in contact with the equipment. Hence, in order to obtain a safe product, it is very crucial to determine the exact time of reaction completion (Vermette et al. 2001).

Monomers used in this study generally contain active acrylic functionalities for free-radical polymerization which are performed in bulk. In this research, methacrylate polyhedral oligomeric silsesquioxane (POSS-acrylate) was introduced and according to our literature review, there are very few reports on the influence of POSS content on the polymerization kinetics of such systems.

Polyhedral oligomeric silsesquioxanes have attracted attention in nanocomposite technology because of inorganic silicate base with organic exterior. The introduction of POSS cages into copolymer backbone results in increased glass transition, decomposition temperature, and oxygen permeability as well as enhanced mechanical properties (Ghasaban et al. 2011; Ke et al. 2012). In our previous study, we also demonstrated an inspiring approach in the synthesis of biomaterials containing POSS cage directly joined to the acrylic-based polymer backbone to improve hardness without sacrificing other essential lens materials properties (Hanifeh et al. 2017). The focus of this present paper is placed on predicting the cure behavior and kinetics of curing reaction of the systems containing multicomponent acrylate-based monomers (hydroxyethyl methacrylate (HEMA), dimethyl itaconate (DMI), methyl methacrylate (MMA), 3-(trimethoxysilyl) propyl methacrylate (TMSPMA) and triethylene glycol dimethacrylate (TGDMA)). The effect of POSS-acrylate introduced into the polymer backbone in free-radical bulk polymerization was studied. Many techniques such as Fourier transform infrared spectroscopy (FTIR), Raman, electron paramagnetic resonance; nuclear magnetic resonance, differential scanning calorimetry (DSC) and differential thermal analysis (DTA) have been used to determine the degree of conversion. Among these, FTIR is the most frequently used method.

Many studies have also focused on the kinetic characterization of thermoset resin systems using DSC (Lam 1989; Ivankovic et al. 2006; Sbirrazzuoli and Vyazovkin 2002; Kamal 1974).

Calorimetry is a widely used method for measuring the reaction rate constants as well as orders of reactions. Here, we report on kinetics study of a multicomponent acrylate-based system, in which a combined curing agents system consisting of triethylene glycol dimethacrylate and POSS was used to render the composition a higher degree of biocompatibility. The reaction rate constants are measured at different temperatures as a function of initiator concentration and the data are compared with those calculated based on Kamal’s model (Kamal 1974).

The results of this research provide insights into our future plan to design suitable lens for treatment of keratoconus which is the most prevalent primary corneal ectasia while the changes in ocular signs and symptoms depend on severity of the disease (Kennedy et al. 1986; Weed et al. 2008; Romero-Jiménez et al. 2010). The disease progress leads to significant loss of vision that cannot be compensated with spectacles. Patients with keratoconus have better vision by semi-rigid contact lenses since these lenses are placed in front of the cornea and pass the light rays to the retina.

## Experiments

### Materials

Hydroxyethyl methacrylate and methyl methacrylate were purchased from Merck, Germany. Triethylene glycol dimethacrylate, dimethyl itaconate, 2,2′-azobisisobutyronitrile (AIBN) and 3-(trimethoxysilyl) propylmethacrylate, were supplied by Sigma-Aldrich, Germany. AIBN was recrystallized in methanol prior to synthesis in order to remove any impurities. Methacrylate polyhedral oligomeric silsesquioxane (POSS-acrylate) was obtained from Hybrid Plastics, USA and used as received. 3-[4,5-Dimethylthiazol-2-yl]-2,5 diphenyltetrazolium bromide MTT, alizarin red staining, dexamethasone and ascorbic acid (Sigma) were purchased from Sigma, UK, Roswell Park Memorial Institute-1640.

### Methods

#### Polymerization reaction

**Fig. 1:**
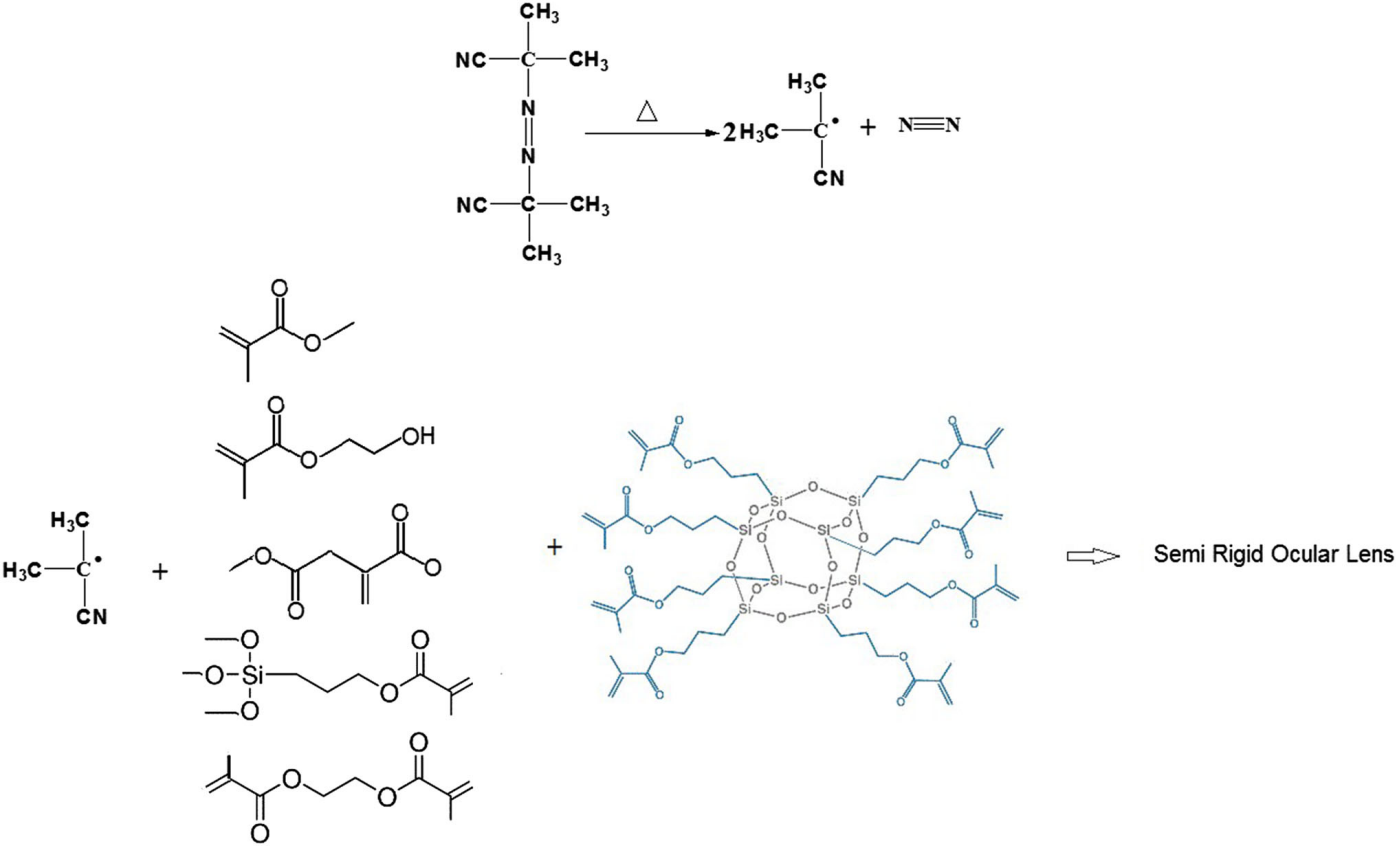
Schematic presentation of polymerization reaction

The synthesis of silicon acrylate lens materials in combination with POSS-acrylate was the objective of this study. The polymerization of a number of carefully selected monomers was carried out taking a free-radical bulk polymerization approach for which the kinetics data as well as degree of conversion is reported here. Therefore, hydroxyethyl methacrylate, methyl methacrylate, triethylene glycol dimethacrylate, dimethyl itaconate, 3-(trimethoxysilyl) propylmethacrylate, and POSS-acrylate mixture were carefully mixed under N_2_ atmosphere until a homogenous mixture was obtained. Then, AIBN was added and the mixture was transferred into a proper mold. In the first step, polymer compounds were heated at optimized temperature (65 °C) for 24 h. The lenses were removed from the mold and maintained at 45 °C for 48 h to complete the polymerization. In Fig. 1, the polymerization reaction is shown schematically.

#### Kinetic study

To study the reaction kinetics as a function of initiator concentration, three formulations were prepared with different concentrations of initiator. The exact composition of each formulation is listed in Table 1.

**Table 1:** Chemical composition of the samples used for kinetics study. The relative concentration of the constituents was kept constant except AIBN, which was used at three different concentrations

	AIBN (g)	TEGDMA (g)	HEMA (g)	POSS (g)	DMI (g)	DMS (g)	NMA (g)
1	0.16	0.2	0.06	0.06	0.3	0.4	0.24
2	0.32	0.2	0.06	0.06	0.3	0.4	0.24
3	0.72	0.2	0.06	0.06	0.3	0.4	0.24

As it is illustrated in Table 1, the concentration of all the constituents was kept constant for three different formulations, except AIBN, which was used at 3 different concentrations of 0.2, 0.4 and 0.6 mol%.

Dynamic DSC experiments were carried out on a Maia 200-F3, Netszch, Germany, at a heating rate of 10 °C/min from ambient temperature until completion of the process in order to determine the total heat of fusion of cure reaction. The exothermic reaction was completed when the heat flow curve versus temperature was leveled off.

Isothermal DSC experiments were also performed on a Maia 200-F3, Netszch, Germany, at four different temperatures of 65, 75, 85 and 95 °C for each formulation.

#### FTIR study

FTIR analysis was carried out to identify the functional groups and degree of polymerization. For this purpose, samples were ground to powder, then mixed with potassium bromide and pressed into discs. To determine the optimal time and temperature in order to complete the polymerization reaction, FTIR spectroscopy was also used.

#### Light absorption test

To measure the transparency of the transmitted light absorption test, a 0.1 mm thick lens was cut. Calibration was performed to measure the amount of visible light passing through a black sheet as a zero-percent reference, and the unobstructed environment between the bulb and the light-absorbing sensor as one hundred-percent reference.

#### Cytotoxicity Evaluation

The cytotoxicity of fabricated lenses was evaluated by extraction method through which the samples were immersed into PBS solution at 37 °C for 28 days. Then, the extraction was applied to the testing system containing L929 fibroblast cells for 24 h and cytotoxicity was studied in accordance with ISO 10993.

The viability of L929 fibroblast cells was also studied using 3-[4,5-dimethylthiazol-2-yl]-2,5-diphenyltetrazolium bromide (MTT). A batch of 100 μL MTT solution with 900 μL medium was incubated with the cells in the wells at 37 °C for 3 h (*n* = 3 for each measurement day). After 3 h, dimethyl sulfoxide solution (DMSO, Sigma Chemical Germany) was added to dissolve formazan crystals. The absorbance values of formazan solutions, obtained from the above given substrates, were measured using an ELIZA reader at 570 nm (Bio-Tek ELx800). All calculations were made using SPSS statistical software. Data are presented as mean ± SD, recorded in triplicate.

## Results and discussion

### Kinetic study

This study also focused on the kinetics of free-radical polymerization of a multicomponent (meth) acrylate system containing variable amounts of thermal initiator. The especial emphasis in this study was focused on the development of a comprehensive kinetics model for free-radical polymerization at initiation stage. Here, kinetic parameters such as activation energy, pre-exponential factor and order of reaction are calculated based on the data obtained from differential scanning calorimetry (DSC), measurements in a set of isothermal experiments especially designed and performed for the sake of kinetics study. When the curing reaction is the only thermal event in the reaction, then the reaction rate or conversion rate must equal the rate of heat flow (*dQ*/*dt*) provided by differential scanning calorimeter.

**Fig. 2:**
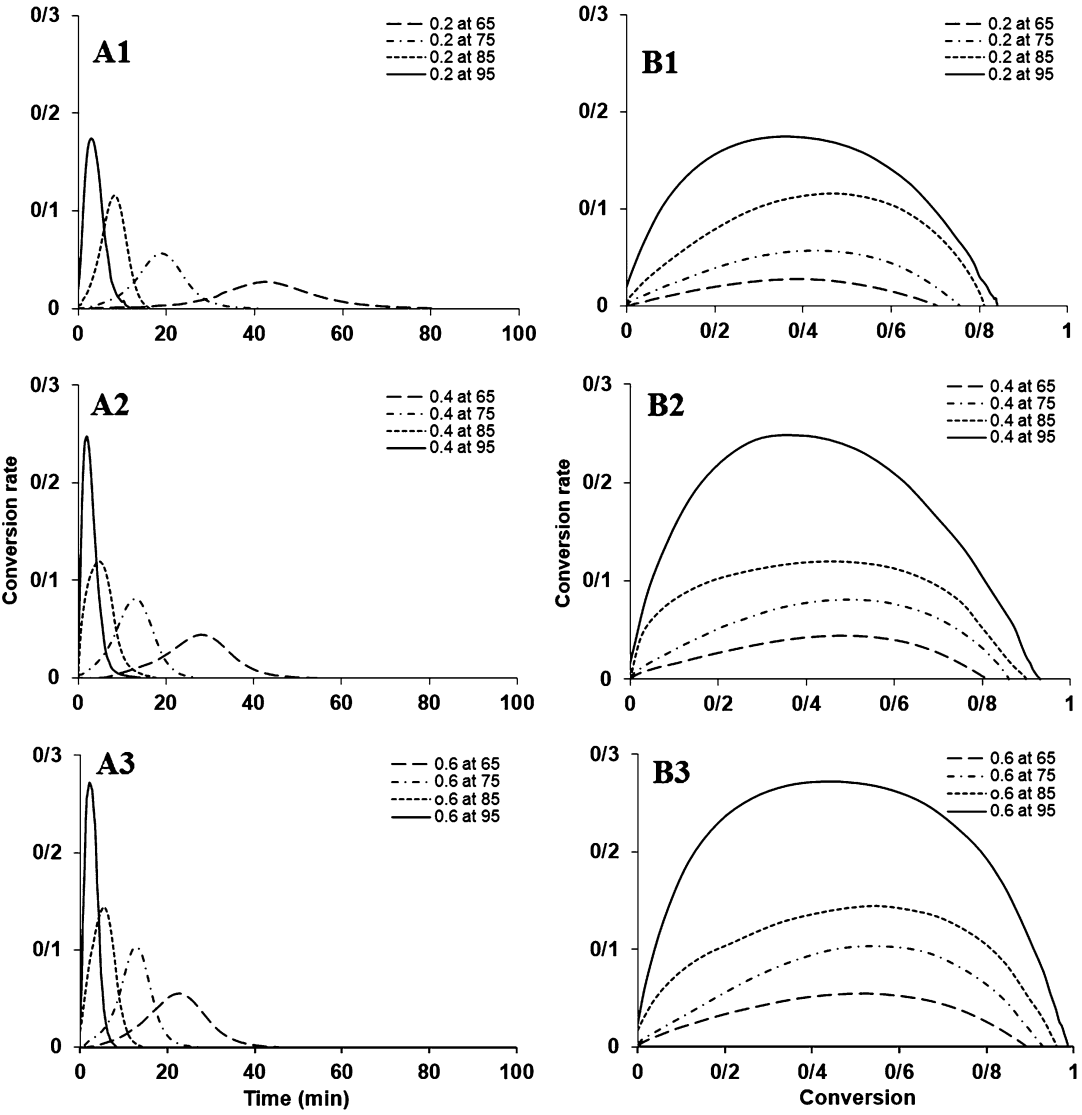
Dependence of the conversion rate of polymeric systems curing time at different isothermal temperatures (the A series) and isothermal reaction rate of the polymeric systems curing as a function of conversion at different isothermal temperatures (the B series)

The values obtained in DSC experiments are used to measure kinetic parameters of the reaction using the equations given in this article. For every isothermal experiment, two lines are drawn from starting and ending points of exothermic peak to the baseline. The area under the curve (AUC), obtained from all the area bounded by the curve, is calculated to measure the heat of reaction. The graphs are divided into smaller parts and the reaction rate, heat of reaction and the heat of the remaining reaction are recorded for each time interval. A fraction is obtained for every area. For kinetic studies by means of DSC, the rate of reaction is supposed to be proportional to the rate of heat generation and can be expressed using Eq. (1) (Kamal 1974; Xu et al. 2017).

1 $$ \frac{d\alpha }{\text{dt}} = \frac{{\frac{dH}{\text{dt}}}}{{\Delta {\text{H}}_{tot} }} $$

where $\frac{d\alpha }{\text{dt}}$ is the rate of the reaction and ΔH_tot_ is the total heat of the reaction. The total heat of reaction generated to reach full conversion, ΔH_tot_, was calculated by DSC dynamic scan at 10 °C/min for each composition. In Fig. 2, the A series, the conversion rate versus time is illustrated for different amounts of initiator at four different temperatures of 65, 75, 85 and 95 °C. Two-phase autocatalytic equation of Kamal was used to calculate the reaction rate constants. Equation 2 shows Kamal’s model (Kamal 1974; Prime 1981; Kenny 1994).

2 $$ \frac{d\alpha }{dt} = (k_{1} + k_{2} \alpha^{m} )(1 - \alpha )^{n} $$

where *n* and *m* are the parameters of the order of reaction; *k*_*1*_, *k*_*2*_ are the reaction rate constants at temperature *T*. The conversion rate is calculated with respect to the time by Eqs. 3 and 4, using the heat flow data obtained from DSC measurements (Khanna and Chanda 1993).

As illustrated in Fig. 2, the B series, the conversion rate is dependent on the reaction temperature and as the polymerization temperature increases, the peak of the conversion rate rises higher and its maximum point moves towards shorter times.

This figure shows the values for the conversion rate with respect to the conversion percentage for different amounts of initiator at 65, 75, 85, 95 °C.

The results reveal that the maximum rate of reaction at 65, 75 and 85 °C has occurred at 50% conversion, while at 95 °C the maximum reaction rate has been registered at about 35–40% conversion.

Reaction rate constants are also temperature dependent and they are calculated by Arrhenius equation (Eq. 3):

3 $$ k_{i} \left( T \right) = Ae^{{\frac{ - E}{RT}}} \,\,\,\,\,\,\,\,\,\,\,\,\,\,\,\,\,\,\,\,\,\,i = 1,2 $$

where *A* is the pre-exponential factor, *E* is the activation energy, *T* is the absolute temperature, and *R* is the universal gas constant. To calculate the unknown values of Eq. 4 in our reaction, this equation is rewritten as a logarithm (Eq. 4)

4 $$ \ln \left[ {\frac{d\alpha }{dt}} \right] = \ln (k_{2} \alpha^{m} + k_{1} ) + n\ln [1 - \alpha ] $$

when Eq. 4 is rewritten, it can be assumed as Eq. 5 or Eq. 6

5 $$ \ln \left[ {\frac{d\alpha }{dt}} \right] - \ln (k_{2} \alpha^{m} + k_{1} ) = n\ln [1 - \alpha ] $$

6 $$ \ln \left\{ {\left[ {\left(\frac{d\alpha }{dt}\right)/(1 - \alpha )^{n} } \right] - k_{1} } \right\} = \ln k_{2} + m\ln \alpha $$

**Fig. 3:**
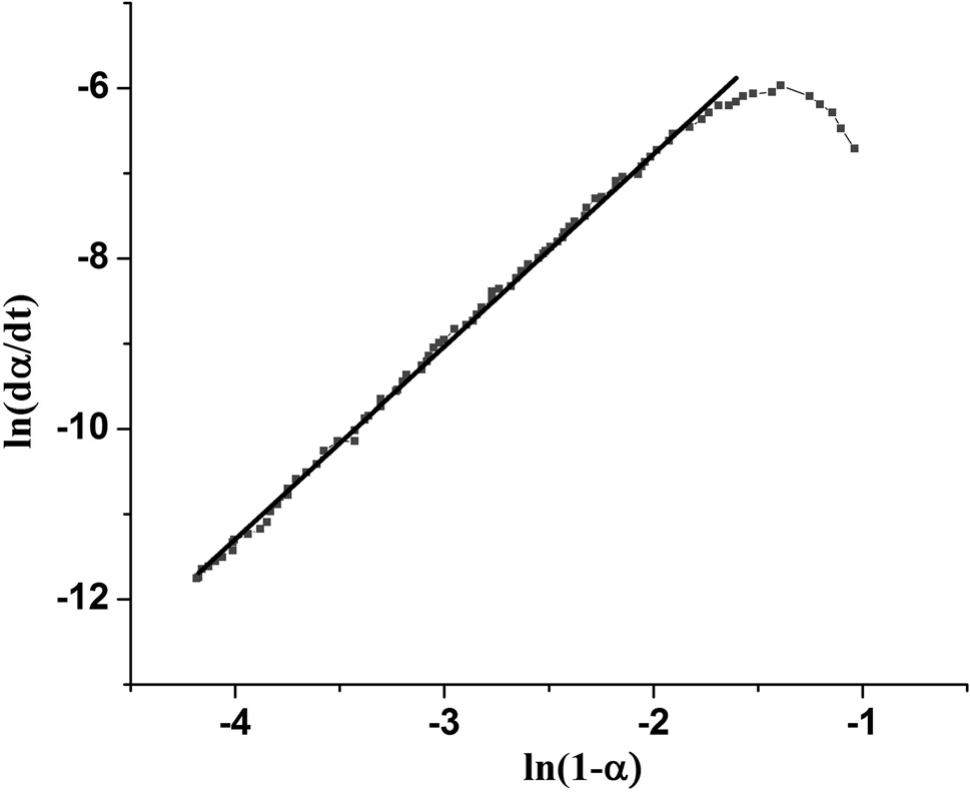
Variation of $\ln \frac{d\alpha }{dt}$ with ln(1–*α*) Slope of the initial linear area of the curve gives an initial estimation for n

The value α at time 0, on α (t) curve plotted versus $\ln \frac{d\alpha }{dt}$, gives K_1_. Graphical representation of Eq. 6 has been illustrated in Fig. 3. The slope of the initial linear part of the curve in Fig. 3 provides the first estimation for n.

**Fig. 4:**
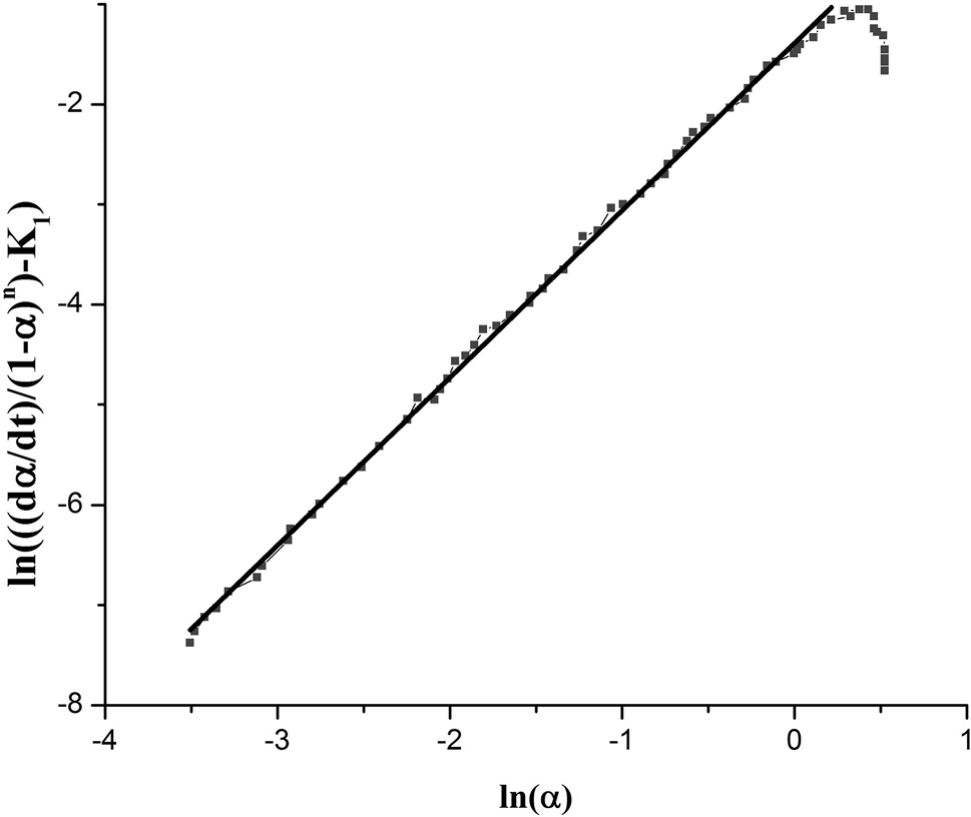
Curve of $\ln \left[ {(\frac{d\alpha }{dt})/(1 - \alpha )^{n} } \right] - k_{1}$ versus ln*α*. From this curve m (slope of the linear part), and lnα (*y*-intercept), are easily calculated

**Fig. 5:**
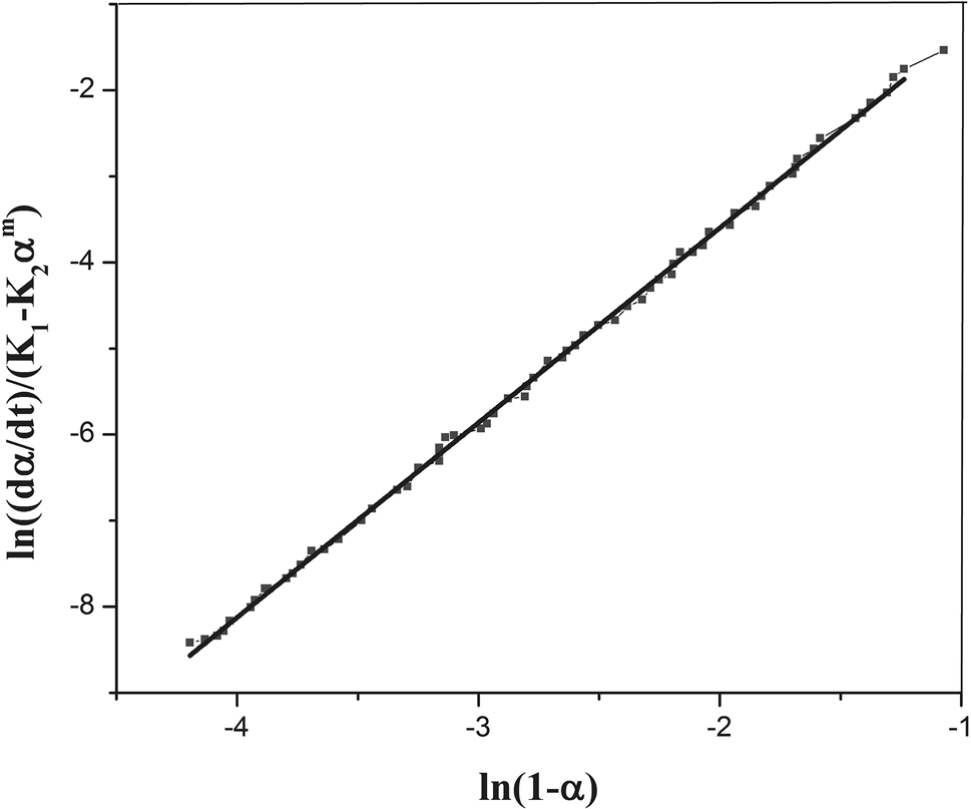
$\ln \left[ {(\frac{d\alpha }{dt})/(k_{2} \alpha^{m} - k_{1} )} \right]$ variation with ln(1–*α*) obtained from Eq. 4 and Eq. 5 for recalculating

Next, the estimated values of *k*_*1*_ and *n* using Eq. (4) are introduced into Eq. 6 by $\ln \left[ {(\frac{d\alpha }{dt})/(1 - \alpha )^{n} } \right] - k_{1}$ plotted versus $\ln \alpha$. The slope of the initial linear part of this curve gives m, while the *y*-intercept is $\ln k_{2}$ (Fig. 4). Having *m*, *k*_*1*_ and *k*_*2*_ values in hand, n is re-calculated from Eq. (5). The *n* obtained this way is different from the initial estimation and all calculations are repeated using the second value estimated for *n*. This continues until the difference between two values is less than one percent. Next, $\ln \left[ {(\frac{d\alpha }{dt})/(k_{2} \alpha^{m} - k_{1} )} \right]$ was plotted against $\ln (1 - \alpha )$ in order to recalculate *n* (Fig. 5).

The reaction rate constant dependence on temperature is given by an Arrhenius type of equation as is shown in Eqs. (1) and (2). The reaction rate constant is calculated using Eq. (7), then

7 $$ k_{1} \left( T \right) = Ae^{{\frac{ - E}{RT}}} \,\,\,\,\,\,\,\,\,\,\,\,\,\,\,\,\,\,\,\,\,\, $$

where *A* is a pre-exponential factor, *E* is the activation energy, *T* is the absolute temperature, and *R* is the universal gas constant. Equation (7) is then rewritten as a logarithm (Eq. 8).

8 $$ \ln \left[ {k\left( T \right)} \right] = \ln \left[ A \right] - \frac{E}{RT} $$

If Eq. 8 is plotted as ln[*k*(*T*)] versus $\frac{1}{T}$, this relation indicates a first degree equation with $\frac{ - E}{R}$ gradient and ln[A] as intercept. The values obtained for reactions rate constants are listed in Table 2 for different concentrations of the initiator.

**Table 2:** Values calcualted for the kinetic parameters of the reaction. n and m are mean values

Initiator (Mol %)	Reaction rate constant	Temperature dependence	*m*	*n*
0.2	K_1_	0	0.92	1.12
	K_2_	4.50 × 10^6^ exp(− 6093/T)		
0.4	K_1_	2.99 × 10^6^ exp(− 8355/T)	0.86	1.12
	K_2_	5.78 × 10^7^ exp(− 7475/T)		
0.6	K_1_	2.49 × 10^5^ exp(− 5446/T)	0.82	1.12
	K_2_	7.75 × 10^6^ exp(− 5798/T)		

**Fig. 6:**
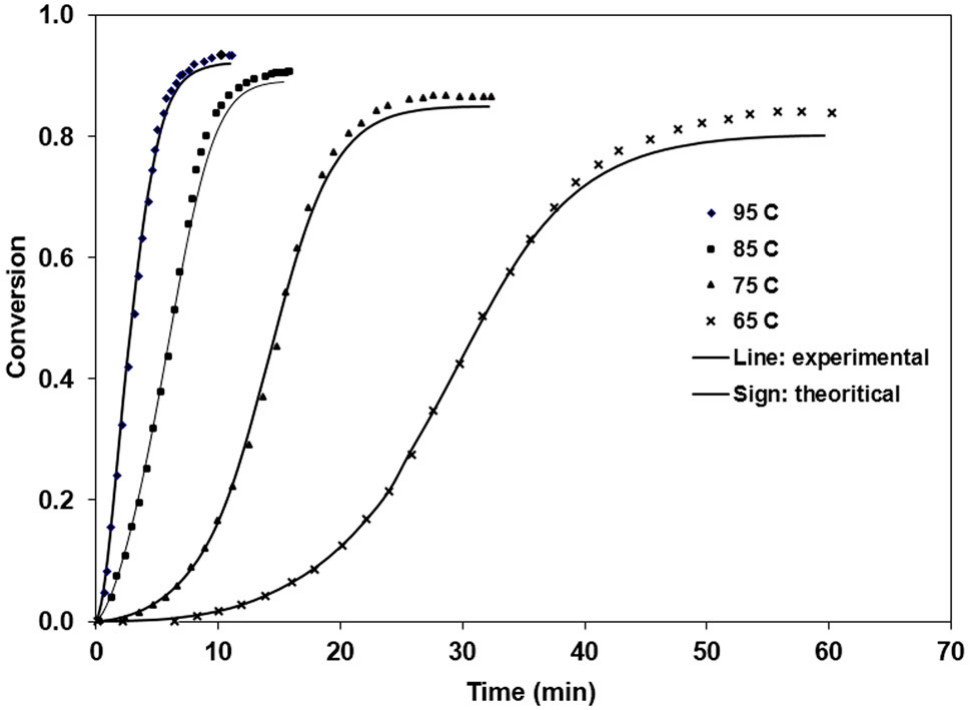
Comparison of experimental conversion data for the polymeric system with the theoretical values calculated based on Kamal’s Model, as a function of time at different isothermal curing temperatures

A comparison between the experimental values obtained from DSC data and the theoretical values estimated based on Kamal’s model is illustrated in Fig. 6. As it can be observed in this figure, there is a good conformity between the estimated values and the experimental values measured here (Hanifeh et al. 2015).

The results in Table 2 show the kinetic rate constants increased with catalyst concentration at the same curing temperature. Meanwhile, the values of each reaction order, m and n, are slightly decreased. Comparisons between the experimental and theoretical cure parameters based on the autocatalytic model show good agreements up to about 80% conversion (where the reaction is kinetically controlled), but beyond that region modest deviations are observed.

### Degree of conversion

The degree of conversion (DC) is determined by measuring the intensity (height or area of absorption band) of unreacted aliphatic C=C double bonds at 1638 cm^−1^ relative to the amount of C=O bonds at 1720 cm^−1^ whose intensity is unaltered during polymerization process [24, 11]. In this technique, it is unnecessary to take into account the sample thickness. Therefore, the percentage of aliphatic C=C bonds remaining unreacted during the polymerization is calculated by the following equation:

9 $$ (\% {\text{DC}}) = 1 - [[{\text{Abs}}({\text{C}}{=}{\text{C}}\;{\text{double}}\;{\text{bonds}})/{\text{Abs}}\;({\text{C}}{=}{\text{O}}\;{\text{bonds}})]\;{\text{polymer}}:\;[{\text{Abs}}\;({\text{C}}{=}{\text{C}}\;{\text{double}}\;{\text{bonds}})/{\text{Abs}}\;({\text{C}}{=}{\text{O}}\;{\text{bonds}})] \times 100 $$

To determine the appropriate reaction temperature and time, the reaction was carried out at temperatures of 65, 75, 85 and 95 °C for 24 h on the sample as mentioned in Table 3. The prepared samples were taken at each individual temperature of infrared range and the reaction progress was examined.

**Table 3:** Degree of conversion of samples synthesized as given in Table 2

AIBN (g)	TEGDMA (g)	HEMA (g)	POSS (g)	DMI (g)	TMSPMA (g)	MMA (g)
0.328	0.312	0.25	0.5	1.5	2.5	1.8

**Fig. 7:**
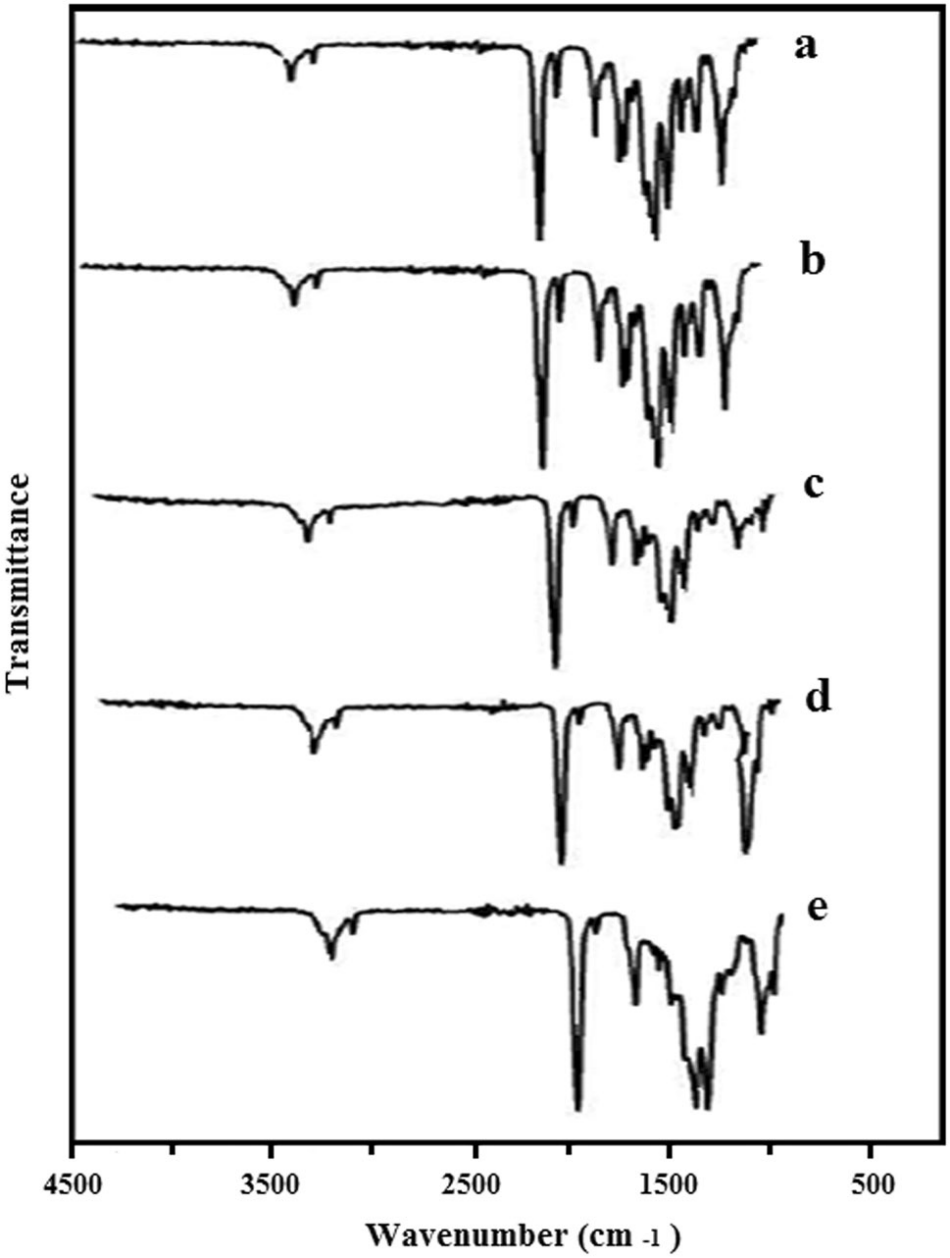
Infrared spectra obtained from synthesized copolymers: **a** before heating and at different temperature **b** 65, **c** 75, **d** 85 and **e** 9 °C for 24 h

In Fig. 7, infrared spectra obtained from synthesized copolymers at different temperatures are shown.

DC calculated at different temperatures from the FTIR spectra is tabulated in Table 4.

**Table 4:** Values calculated for kinetic parameters of the reaction. *n* and *m* are mean values

Reaction temperature (°C)	65	75	85	95
Degree of conversion DC (%)	55	69	84	96

The degree of conversion calculation shows that by increasing the temperature, the DC increases which could be due to rapid formation of free radicals, increasing the number of effective monomer collisions and increasing the number of monomers with sufficient energy for reaction.

### Light absorption test

To evaluate the transparency of the lenses, the light absorption test has been carried out. The light passage rate for this sample is more than 92% the same as light passage rate for pure methyl methacrylate (92%). Therefore, the manufactured specimen has a suitable light passage rate for use as a transparent material.

### Cytotoxicity test

**Fig. 8:**
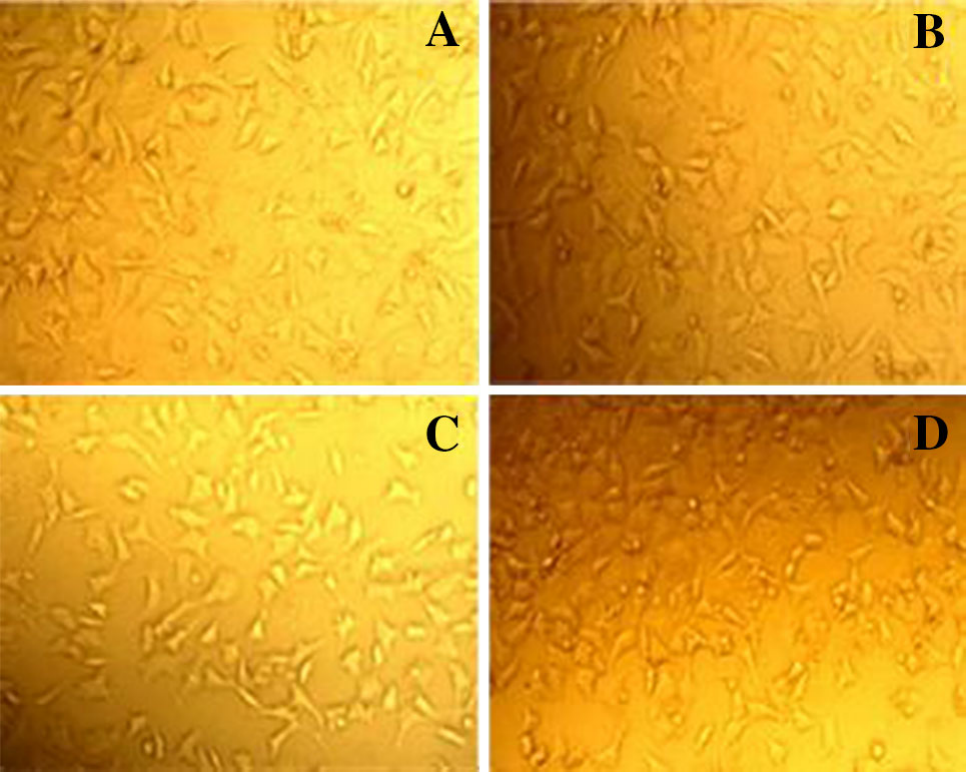
Light microscope images of L929 fibroblast cell culture in extracts from contact lenses. **a** control **b** synthesized at 50 °C, **c** synthesized at 60 °C and **d** synthesized at 70 °C for 24 h

**Fig. 9:**
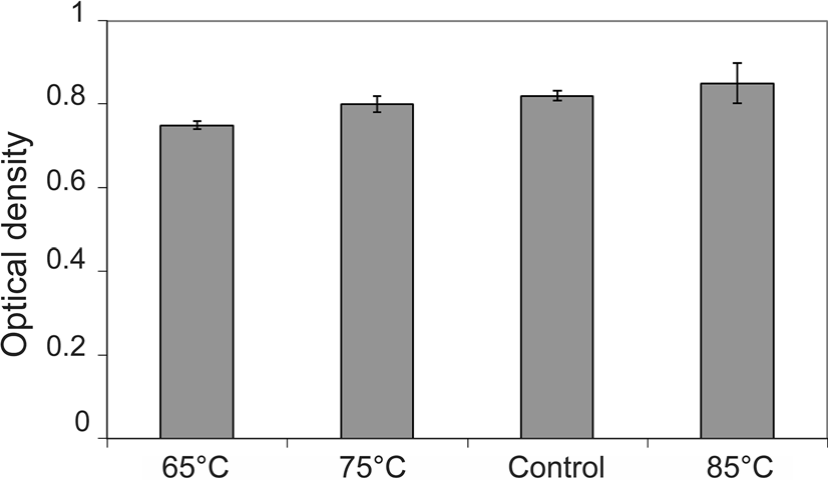
Cell viability versus synthesizing temperature: **a** cell viability of L929 fibroblast cells tested at 50 °C, 60 °C and 70 °C in compared to tissue culture plate as control from MTT assay. Data are expressed as mean ± SD (*n* = 3). Data are subject to Dunnet one-way analysis of variance (ANOVA), ***p < 0.001

L929 fibroblast cell culturing was used to confirm that fabricated lenses have no toxicity impact on the fibroblast cells. Therefore, lenses synthesized in different temperatures were defined for culturing the fibroblast cells. The cells were cultured in the extract from lenses synthesized at 50, 60 and 70 °C. As it is illustrated in Fig. 8, the cells grow and proliferate into the extract and it shows no toxic substance into the extraction media (Fig. 9).

MTT assay which is sensitive to detect small differences in cell number was used on the lens materials to analyze fibroblast cells growth rate. With regard to fibroblast cells behavior in vicinity of the lenses, the results demonstrate some minor changes in cell number. According to these results, lenses synthesized at 65-85 °C show less toxicity.

## Conclusion

For synthesis of silicone acrylate-based lens materials containing POSS, a free-radical bulk polymerization was chosen. In this study, curing reaction kinetics was investigated by means of two techniques: differential scanning calorimetry and FTIR spectroscopy. Kinetics parameters of the polymerization systems specially designed for contact lens formulations were estimated based on Kamal’s autocatalytic model. The results from DSC experiments revealed that the reaction rate increased with temperature for all copolymers and that peak reaction rate in reaction rate versus time curve moved towards shorter times. Moreover, the maximum rate of reaction for the reactions at temperatures 65, 75 and 85 °C has occurred at conversion levels of 70–40%, while the maximum rate of reaction at 95 °C occurred at about 35% conversion. These results clearly showed that as the curing temperature was increased, the system exhibited better agreement with the Kamal’s autocatalytic model and the best agreement was observed for the system cured at 95 °C.

The completion in reaction of the multicomponent systems containing POSS was studied with FTIR spectroscopy, for which the results showed a perfect match with the results of DSC experiments (complete removal of the C=C vibration at 1638 cm^−1^ in the FTIR spectrum at 95 °C).

Cytotoxicity tests showed that the growth and proliferation of cells within the extracted media are comparable with the cells growth and proliferation in the culture medium (as control).

## References

[CR1] Ghasaban S, Atai M, Imani M, Zandi M, Shokrgozar MA (2011). Photo-crosslinkable cyanoacrylate bioadhesive: shrinkage kinetics, dynamic mechanical properties, and biocompatibility of adhesives containing TMPTMA and POSS nanostructures as crosslinking agents. J Biomed Mater Res Part A.

[CR2] Hanifeh M, Zandi M, Shokrollahi P, Ataei M, Ghafarzadeh E, Shokrolahi F (2015) Fabrication of multicomponent silicone acrylate based copolymer utilized in ocular lenses. International Conference and exhibition on advanced and nano materials

[CR3] Hanifeh M, Zandi M, Shokrollahi P, Ataie M, Ghafarzadeh E, Askari F (2017). Compositional design and taguchi optimization of hardness properties in silicone based ocular lenses. Prog Biomater.

[CR4] Ivankovic M, Brnardic I, Ivankovic H, Mencer HJ (2006). DSC study of the cure kinetics during nanocomposite formation: epoxy/poly (oxypropylene) diamine/organically modified montmorillonite system. J Appl Polym Sci.

[CR5] Kamal MR (1974). Thermoset characterization for moldability analysis. Polym Eng Sci.

[CR6] Ke F, Zhang C, Guang S, Xu H (2012). POSS Core star-shape molecular hybrid materials: effect of the chain length and POSS content on dielectric properties. J Appl Polym Sci.

[CR7] Kennedy RH, Bourne WM, Dyer JA (1986). A 48-year clinical and epidemiologic study of Keratoconus. Am J Ophthalmol.

[CR8] Kenny JM (1994). Determination of autocatalytic kinetic model parameters describing thermoset cure. J Appl Polym Sci.

[CR9] Khanna U, Chanda M (1993). Kinetics of anhydride curing of isophthalic diglycidyl ester using differential scanning calorimetry. J Appl Polym Sci.

[CR10] Lam PWK (1989). The characterization of thermoset cure behavior by differential scanning calorimetry. Part II: effects of low-profile additives. Polymer Compos.

[CR11] Lee JH, Lee JW (1994). Kinetic parameters estimation for cure reaction of epoxy based vinyl ester resin. Polym. Engin. Sci..

[CR12] Mannis MJ, Zadnik K, Coral-Ghanem C, Kara-José N (2003). Contact lenses in ophthalmic practice.

[CR13] Peinado C, Salvador AF, Basega J, Catalina F (2002). Following in situ photoinitiated polymerization of multifunctional acrylic monomers by fluorescence and photocalorimetry simultaneously. Polymer.

[CR14] Prime RB (1981) Thermal characteristics of polymeric materials. In E Turi (Ed) Academic Press, New York

[CR15] Rathi VM, Mandathara PS, Dumpati S (2011). Boston ocular surface prosthesis: an Indian experience. Ind J Ophth.

[CR16] Romero-Jiménez M, Santodomingo-Rubido J, Wolffsohn JS (2010). Keratoconus: a review. Contact Lens Anterior Eye.

[CR17] Sbirrazzuoli N, Vyazovkin S (2002). Learning about epoxy cure mechanisms from isoconversional analysis of DSC data. Thermochim Acta.

[CR18] Vermette P, Griesser HJ, Laroche G, Guidoin R (2001). Biomedical application of polyurthane. Tissue Eng Intell.

[CR19] Vinnik M, Roznyatovsky VA (2004). Kinetic method by using calorimetry to mechanism of epoxy-amine cure reaction. J Therm Anal Cal.

[CR20] Wang W, Hutchinson RA (2010). Free-radical acrylic polymerization kinetics at elevated temperatures. Chem Eng Technol.

[CR21] Weed KH, MacEwen CJ, Giles T, Low J, McGhee CN (2008). The dundee University Scottish Keratoconus study: demographics, corneal signs, associated diseases, and eye rubbing. Eye.

[CR22] Woo EM, Seferis JC (1990). Cure kinetics of epoxy/anhydride thermosetting matrix systems. J Appl Polym Sci.

[CR23] Xu X, Zhou Q, Song N, Ni Q, Ni L (2017). Kinetic analysis of isothermal curing of unsaturated polyester resin catalyzed with tert-butyl peroxybenzoate and cobalt octoate by differential scanning calorimetry.

