# Correction to: Cure kinetic study of methacrylate-POSS copolymers for ocular Lens

**DOI:** 10.1007/s40204-018-0081-6

**Published:** 2018-01-23

**Authors:** F. Shokrolahi, M. Zandi, P. Shokrollahi, M. Atai, E. Gafar-Zadeh, M. Hanifeh

**Affiliations:** 1 Biomaterials Department, Iran Polymer and Petrochemical Institute, Pazhoohesh Blvd, Tehran-Karaj Hwy, 1497713115 Tehran, Iran; 2 Science Department, Iran Polymer and Petrochemical Institute, Pazhoohesh Blvd, Tehran-Karaj Hwy, 1497713115 Tehran, Iran; 3 Lassonde School of Engineering, York University, 4700 Keele Street, 11 Arboretum Ln, ON M3J 1P3 Toronto, Canada

## Correction to: Prog Biomater (2017) 6:147–156 10.1007/s40204-017-0074-x

The original version of this article unfortunately contained a mistake: the spelling of the Ebrahim Gafar-Zadehs’ name was incorrect. The corrected name is given above.

